# Supplementary material for: Cross-species experiments reveal widespread cochlear neural damage in normal hearing
Source: Commun Biol. 2022 Jul 22;5:733. doi: 10.1038/s42003-022-03691-4 (PMC9307777; doi:10.1038/s42003-022-03691-4)
Supplement: Supplementary file 1 — Supplementary Information [file 42003_2022_3691_MOESM1_ESM.pdf]

Cross-Species Experiments Reveal Widespread Cochlear Neural Damage in Normal Hearing

*Hari M. Bharadwaj[a,b], Alexandra R. Hustedt-Mai[a], Hannah M. Ginsberg[b], Kelsey M. Dougherty[a], Vijaya Prakash Krishnan Muthaiah[c], Anna Hagedorn[a], Jennifer M. Simpson[a], and Michael G. Heinz[a,b]

[a] *Department of Speech, Language, and Hearing Sciences, Purdue University, West Lafayette, IN, United States*

[b] *Weldon School of Biomedical Engineering, Purdue University, West Lafayette, IN, United States*

[c] *Department of Rehabilitation Sciences, University at Buffalo, Buffalo, NY, United States*

*Correspondence: `hbharadwaj@purdue.edu`

Supplementary Information

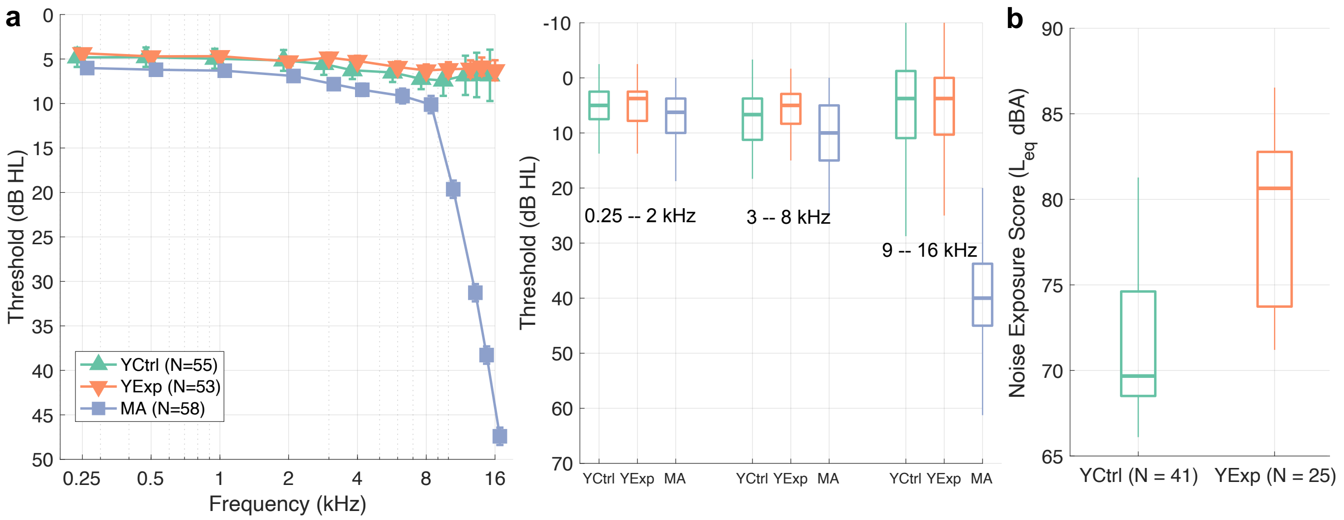

Supplementary Figure 1. a Audiometric thresholds show tight matching between groups in the standard clinical frequency range (up to 8 kHz). YCtrl and YExp groups are tightly matched up to 16 kHz. Data points on the left side of panel A represent mean ± STE bars. Box plots of audiometric thresholds averaged over three separate frequency ranges (0.25 – 3 kHz, 3 – 8 kHz, and 9 – 16 kHz) are shown on the right side of Panel A to visualize the full distribution of the data. **b** As expected, noise-exposure scores from a subset of young controls (YCtrl) and young noise-exposed subjects (YExp) confirmed that the two are well-separated in their reporting of acoustic exposures. All box plots show the median line enclosed in a box denoting the 25th to 75th percentile range, and whiskers of length 1.5 times the interquartile range. Underlying data are archived on Zenodo (doi: 10.5281/zenodo.6672827).

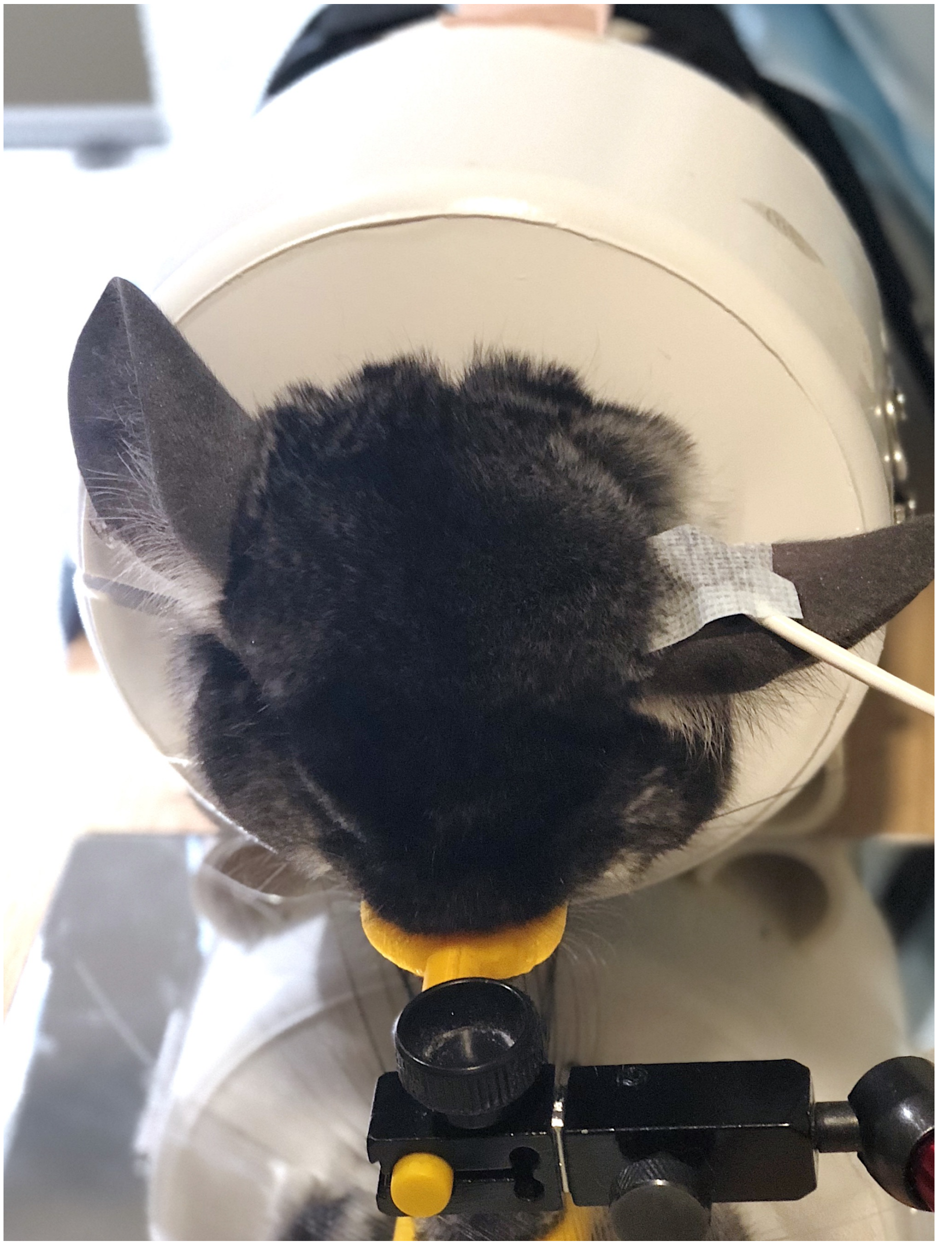

Supplementary Figure 2. Photograph of awake chinchilla setup for DPOAE and WB-MEMR measurements.

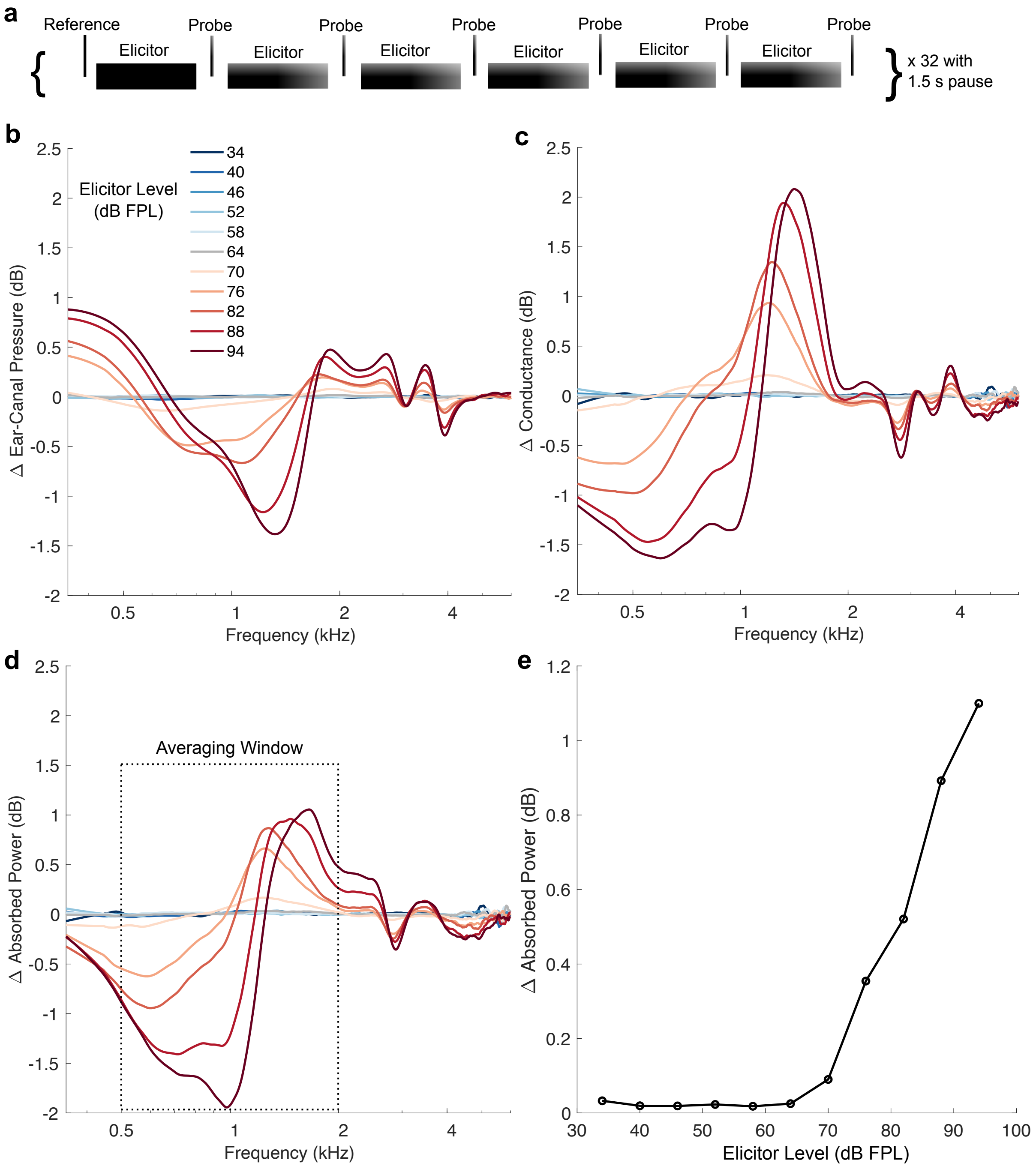

Supplementary Figure 3. WB-MEMR measurement paradigm and example data from a human participant. **a** Trial sequence consisting of alternate click probe and ipsilateral elicitors. **b** Frequency pattern of WB-MEMR immittance change quantified as change in ear-canal pressure for the probe click. **c** Conductance change associated with MEMR. **d** WB-MEMR immittance change quantified as change in absorbed power, i.e., sum of values in (**b**) and (**c**). **e** Raw (before baseline correction and sigmoid curve fitting) WB-MEMR growth function with elicitor level extracted by averaging absolute dB-changes in absorbed power in the 0.5–2 kHz range.

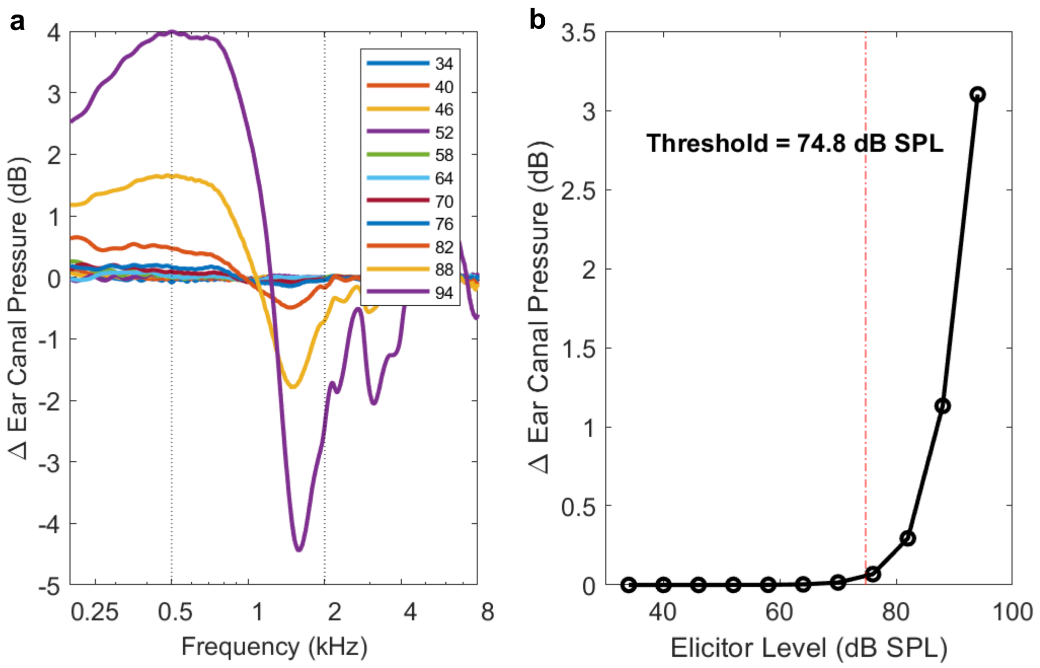

Supplementary Figure 4. WB-MEMR example from a chinchilla participant (pre-NE). **a** Stereotypical alternating profile of WB-MEMR response across frequency. **b** Fitted WB-MEMR growth function with elicitor level extracted by averaging absolute dB-changes in ear-canal pressure in the 0.5 – 2 kHz range and fitting a sigmoid growth function.

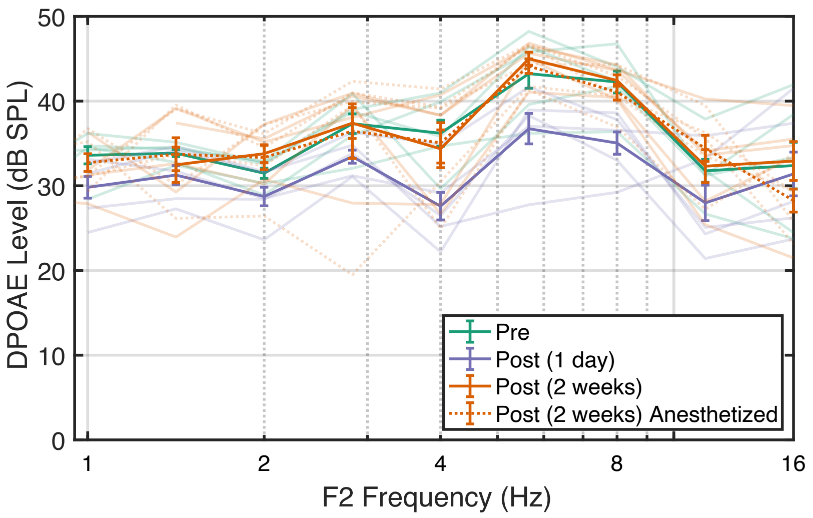

Supplementary Figure 5. DPOAEs measured using 75/65 dB SPL pure-tone primaries instead of the swept-tone protocol. Response values are comparable to swept-tone measurements in Fig. 1(textbfc). Data points represent mean ± STE bars (N=5). Measurements from individual subjects are shown as transparent lines color coded by whether the values were obtained pre-, 1-day post-, or 2-weeks post-NE. Underlying data are archived on Zenodo (doi: 10.5281/zenodo.6672827).

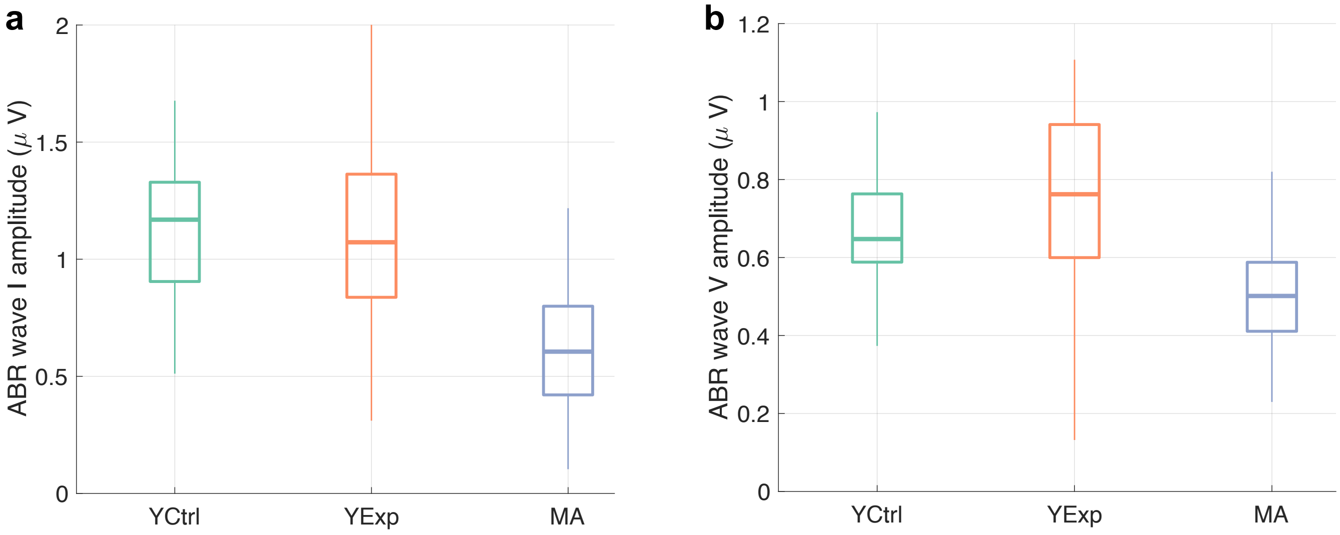

Supplementary Figure 6. Separate wave I (**a**) and wave V (**b**) values shown for the three human groups: YCtrl (N=55), YExp (N=53), and MA (N=58). The ABR wave I/V ratio derived from these values was used as a putative assay of cochlear synaptopathy in Fig. 2(**c**). All box plots show the median line enclosed in a box denoting the 25th to 75th percentile range, and whiskers of length 1.5 times the interquartile range. Underlying data are archived on Zenodo (doi: 10.5281/zenodo.6672827).

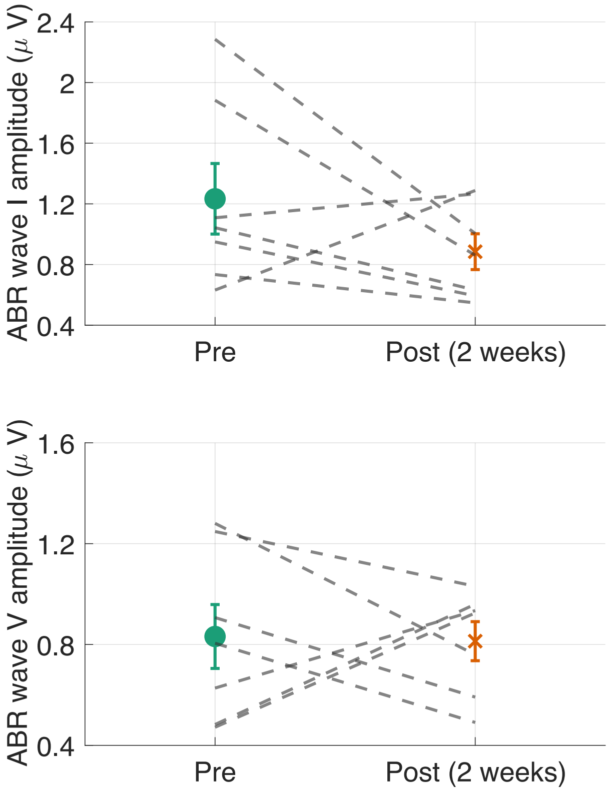

Supplementary Figure 7. Separate wave I (top) and wave V (bottom) values shown for chinchilla subjects. The ABR wave I/V ratio derived from these values was used as a putative assay of cochlear synaptopathy in Fig. 1(**f**). Data points represent mean ± STE bars (N=7). Measurements from individual subjects are shown as dotted gray lines. Underlying data are archived on Zenodo (doi: 10.5281/zenodo.6672827).

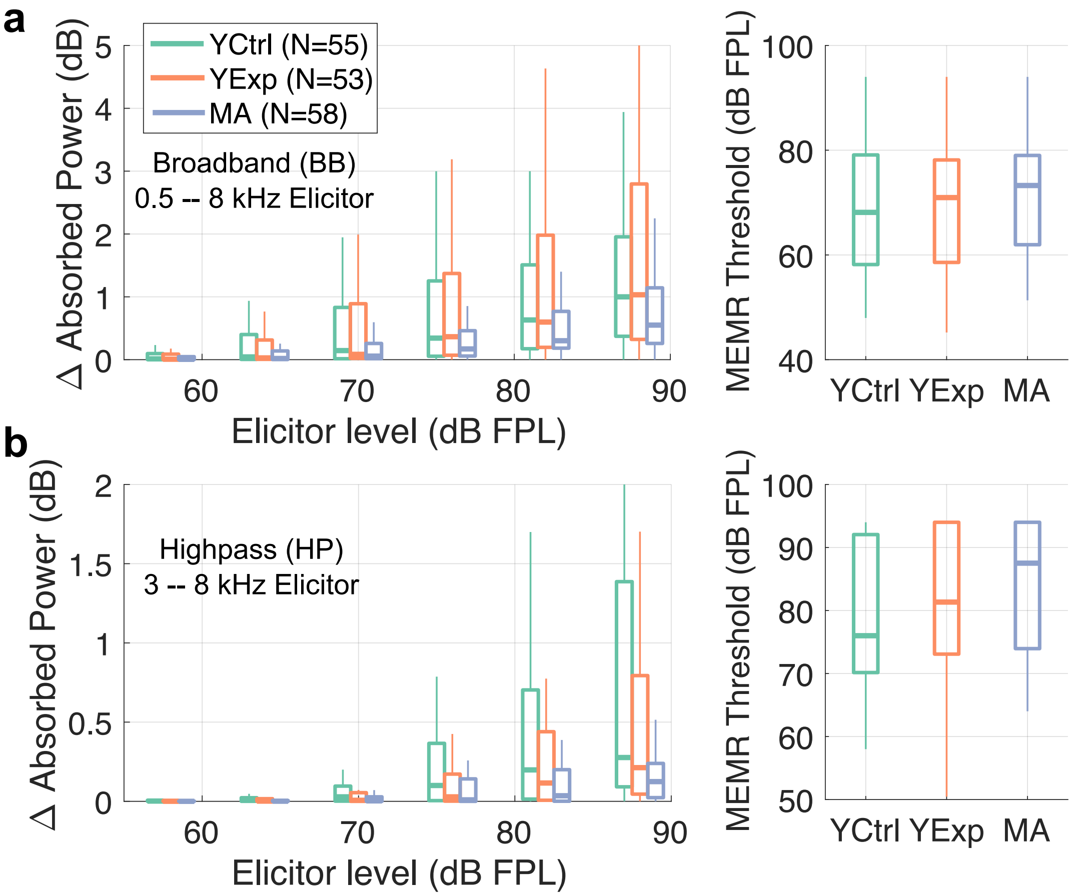

Supplementary Figure 8. **a** Box plots of WB-MEMR data from human subjects previously summarized in Fig. 2(**b**) are shown to help visualize the full distribution of the data at high BB elicitor levels. **b** Box plots of WB-MEMR data from human subjects previously summarized in Fig. 2(**d**) are shown to help visualize the full distribution of the data at high HP elicitor levels. All box plots show the median line enclosed in a box denoting the 25th to 75th percentile range, and whiskers of length 1.5 times the interquartile range. Underlying data are archived on Zenodo (doi: 10.5281/zenodo.6672827).
