# Supplementary material for: Cross-species experiments reveal widespread cochlear neural damage in normal hearing
Source: Commun Biol. 2022 Jul 22;5:733. doi: 10.1038/s42003-022-03691-4 (PMC9307777; doi:10.1038/s42003-022-03691-4)
Supplement: Supplementary file 2 — Life Sciences Reporting Summary [file 42003_2022_3691_MOESM2_ESM.pdf]

nature research

Corresponding author(s): Hari Bharadwaj

Last updated by author(s): Jun 1, 2022

Reporting Summary

Nature Research wishes to improve the reproducibility of the work that we publish. This form provides structure for consistency and transparency in reporting. For further information on Nature Research policies, see our Editorial Policies and the Editorial Policy Checklist.

Statistics

For all statistical analyses, confirm that the following items are present in the figure legend, table legend, main text, or Methods section.

n/a	Confirmed	
☐	☒	The exact sample size (*n*) for each experimental group/condition, given as a discrete number and unit of measurement
☐	☒	A statement on whether measurements were taken from distinct samples or whether the same sample was measured repeatedly
☐	☒	The statistical test(s) used AND whether they are one- or two-sided<br>*Only common tests should be described solely by name; describe more complex techniques in the Methods section.*
☐	☒	A description of all covariates tested
☐	☒	A description of any assumptions or corrections, such as tests of normality and adjustment for multiple comparisons
☐	☒	A full description of the statistical parameters including central tendency (e.g. means) or other basic estimates (e.g. regression coefficient) AND variation (e.g. standard deviation) or associated estimates of uncertainty (e.g. confidence intervals)
☐	☒	For null hypothesis testing, the test statistic (e.g. *F*, *t*, *r*) with confidence intervals, effect sizes, degrees of freedom and *P* value noted<br>*Give P values as exact values whenever suitable.*
☒	☐	For Bayesian analysis, information on the choice of priors and Markov chain Monte Carlo settings
☒	☐	For hierarchical and complex designs, identification of the appropriate level for tests and full reporting of outcomes
☐	☒	Estimates of effect sizes (e.g. Cohen's *d*, Pearson's *r*), indicating how they were calculated

Our web collection on statistics for biologists contains articles on many of the points above.

Software and code

Policy information about availability of computer code

Data collection	Acoustic measurements were done using custom software in MATLAB. This software is publicly available at https://github.com/SNAPsoftware/SNAPacoustics. The license is minimally restrictive; investigators interested in replicating the measures used in this study can adapt the code to match the specifics of their hardware. Electrophysiological measurements were acquired using software provided by the EEG system manufacturer (Biosemi).
Data analysis	Custom software used for data analysis are publicly available at at https://github.com/SNAPsoftware/ANLffr and https://github.com/SNAPsoftware/SNAPacoustics. Statistical analysis was done using the LME4 package in R.

For manuscripts utilizing custom algorithms or software that are central to the research but not yet described in published literature, software must be made available to editors and reviewers. We strongly encourage code deposition in a community repository (e.g. GitHub). See the Nature Research guidelines for submitting code & software for further information.

Data

Policy information about availability of data

All manuscripts must include a data availability statement. This statement should provide the following information, where applicable:

- Accession codes, unique identifiers, or web links for publicly available datasets
- A list of figures that have associated raw data
- A description of any restrictions on data availability

Individual human subject WB-MEMR responses for two different elicitors as a function of elicitor level, ABR wave I and V amplitudes, audiometric thresholds binned in three different frequency ranges, DPOAE amplitudes in the 3-8 kHz range and the 8-16 kHz range, age and gender can be obtained from https://github.com/haribharadwaj/CommunBiol_CrossSpecies_Synaptopathy and are permanently archived using Zenodo at https://doi.org/10.5281/zenodo.6672827. Similarly, WB-

MEMR, ABR, and OAE data for individual chinchillas at the pre- and 2-week-post-NE time points can be obtained from the same repository. The NHANES 2011-2012 audiological data is publicly available at https://www.cdc.gov/nchs/nhanes/.

Field-specific reporting

Please select the one below that is the best fit for your research. If you are not sure, read the appropriate sections before making your selection.

☒ Life sciences ☐ Behavioural & social sciences ☐ Ecological, evolutionary & environmental sciences

For a reference copy of the document with all sections, see nature.com/documents/nr-reporting-summary-flat.pdf

Life sciences study design

All studies must disclose on these points even when the disclosure is negative.

Sample size

Sample size of 50 per group for the human comparisons was guided by observed effect sizes (correlation coefficients) in previous studies comparing physiological measurements from the early portions of the auditory pathway and behavior. This number was also appropriate to obtain a high specificity (95%) and sensitivity (80%) of detecting medium between-group effect sizes (Cohen's d of 0.5-0.6). A medium effect size is clinically significant in audiology, and also significant as a source of variance in auditory neuroscience data. For chinchilla data, because a pre-post within-animal design is used, N=6-8 was considered a good balance between obtaining adequate power while minimizing the use of animals for research.

Data exclusions

No data were excluded from statistical analysis.

Replication

Rigorous methods were employed to estimate the effect sizes and the variance of the estimated effect sizes. The primary effects obtained using targeted lab-based assays are medium-large for individual metrics, and large for hybrid metrics constructed with full battery. With clinic-style measures, the results were replicated but with smaller effects sizes as expected from their less targeted nature. The effect of age-related declines in MEMR were further replicated by comparing to a large publicly available dataset from the N-HANES repository.

Randomization

The present study does not perform a cross-sectional intervention. With human subjects, it probes the differences in inner ear physiology between groups that differ in age or noise-exposure history but otherwise tightly matched in demographics characteristics. Thus random assignment is not relevant for the human comparisons. The order in which data were acquired from different participants was random driven by the logistics of subject recruitment. For chinchilla data too, the comparisons were within-animal pre- vs. post-noise-exposure and not across groups of animals. Hence randomization is not relevant for the animal data.

Blinding

Randomization was not relevant for group allocation. For data analysis, automated scripts were used to quantify the effects that are reported as significant. These were the WB-MEMR differences in both humans and chinchillas, and the ABR differences in human groups. For replication experiments with clinic-style measures, automated scripts were used for MEMR quantification. However, blinding was not done for ABR peak picking with the clinic-style ABR measure, and instead done by trained research assistants who were also audiology graduate students whose clinical training includes manual peak picking. The manual peak-picking approach was chosen for the clinic-style measure to imitate standard clinical procedures.

Reporting for specific materials, systems and methods

We require information from authors about some types of materials, experimental systems and methods used in many studies. Here, indicate whether each material, system or method listed is relevant to your study. If you are not sure if a list item applies to your research, read the appropriate section before selecting a response.

Materials & experimental systems

n/a	Involved in the study	
☒	☐	Antibodies
☒	☐	Eukaryotic cell lines
☒	☐	Palaeontology and archaeology
☐	☒	Animals and other organisms
☐	☒	Human research participants
☒	☐	Clinical data
☒	☐	Dual use research of concern

Methods

n/a	Involved in the study	
☒	☐	ChIP-seq
☒	☐	Flow cytometry
☒	☐	MRI-based neuroimaging

Animals and other organisms

Policy information about studies involving animals; ARRIVE guidelines recommended for reporting animal research

Laboratory animals

Young (under 1.5 years of age) male genetically heterogenous (i.e., wild-type) chinchillas weighing 400 to 650 grams were used in the study.

Wild animals

Study did not involve wild animals

Field-collected samples	Study did not involve samples collected in the field
Ethics oversight	Animals were used in accordance with protocols approved by the Purdue Animal Care and Use Committee: PACUC Protocol No: 1111000123.

Note that full information on the approval of the study protocol must also be provided in the manuscript.

Human research participants

Policy information about studies involving human research participants

Population characteristics	The participants in the three human groups were matched in audiometric hearing sensitivity in the standard clinically tested range given that the hypotheses specifically focused on sub-clinical damage in the inner ear among individuals with matched hearing sensitivity. Subjects were recruited without reference to sex, gender, race or ethnicity and the cohorts reflected the balance of demographic characteristics in the local community. The gende-split is reported in the Methods section. Gender was used as a covariate in the statistical analysis such that all reported effects reflect the average across genders.
Recruitment	Participants were recruited through community sources using posted flyers and advertizements on bulleting boards. Furthermore, because the study compares sensory processing between human groups using passively measured neurophysiological responses, we do not anticipate any effects of selection bias.
Ethics oversight	All human subject measures were conducted in accordance with protocols approved by the Purdue University Internal Review Board and the Human Research Protection Program: IRB Protocol No. 1609018209.

Note that full information on the approval of the study protocol must also be provided in the manuscript.
